# The Effect of Tapered Abutments on Marginal Bone Level: A Retrospective Cohort Study

**DOI:** 10.3390/jcm8091305

**Published:** 2019-08-24

**Authors:** Simone Marconcini, Enrica Giammarinaro, Ugo Covani, Eitan Mijiritsky, Xavier Vela, Xavier Rodríguez

**Affiliations:** 1 Department of Surgical, Medical, Molecular and Critical Area Pathology, University of Pisa, 56124 Pisa, Italy; 2 Tuscan Dental Institute, Versilia General Hospital, 55041 Lido di Camaiore, Italy; 3 Department of Otolaryngology Head and Neck Surgery and Maxillofacial Surgery, Tel-Aviv Sourasky Medical Center, Sackler School of Medicine, 61503 Tel Aviv, Israel; 4 Department of Maxillofacial Surgery and Implantology, International University of Catalunya, 08001 Barcelona, Spain; 5 Department of Oral Implantology, European University of Madrid, 28001 Madrid, Spain

**Keywords:** bone loss, convergence, clinical study

## Abstract

Background: Early peri-implant bone loss has been associated to long-term implant-prosthetic failure. Different technical, surgical, and prosthetic techniques have been introduced to enhance the clinical outcome of dental implants in terms of crestal bone preservation. The aim of the present cohort study was to observe the mean marginal bone level around two-part implants with gingivally tapered abutments one year after loading. Methods: Mean marginal bone levels and change were computed following radiological calibration and linear measurement on standardized radiographs. Results: Twenty patients who met the inclusion criterion of having at least one implant with the tapered prosthetic connection were included in the study. The cumulative implant success rate was 100%, the average bone loss was −0.18 ± 0.72 mm, with the final bone level sitting above the implant platform most of the time (+1.16 ± 0.91 mm). Conclusion: The results of this cohort study suggested that implants with tapered abutments perform successfully one year after loading and that they are associated with excellent marginal bone preservation, thus suggesting that implant-connection macro-geometry might have a crucial role in dictating peri-implant bone levels.

## 1. Introduction

Long-term dental implant survival has been extensively documented under different conditions, so that contemporary clinical dentistry has been focusing on means to achieve predictable implant success. Most of the authors agree on the fact that minimal marginal bone loss should be observed one year within the implant loading, as this quantity is a predictor of the long-term implant survival and success [1]. The extent of post-loading bone remodeling has been mainly related to two different phenomena: (1) The microbial infiltration at the implant-abutment (IA) micro-gap—with consequent inflammation and bone demineralization [2]; (2) the implant-abutment (IA) design [3].

The most accounted risk factor for marginal bone loss has been long considered the inflammatory infiltrate at the IA gap [4]. The understanding of the complex biological events impacting the cervical bone surrounding submerged implants begun with the fundamental animal histometric study by Ericsson [5] who typified the inflammatory infiltrate as a consistent finding in matching IA interfaces. This circumscribed inflammation resulted in a round connective demarcation wall that ultimately leads to bone demineralization and resorption [2,6,7]. Different studies indicated less marginal bone resorption around mis-matching implants (implants with a platform switching connection—PS)—when compared to matching implants—as well as a different organization of the connective tissue fibers [8]. Several theories have been proposed to explain this clinical manifestation, such as the shifting of the inflammatory infiltrate away from the bone, the additional room for protective connective tissue proliferation, or, best, the creation of a geometrical stop for biological width apical establishment. In fact, in matching implants, the fixture first thread is also the first topographic point where the rehabilitation turns from a smaller to a wider diameter, creating a mechanical retention for connective tissues. In short, marginal bone loss should be inevitable, at least to this extent [9]. In PS implants, the implant-abutment discrepancy acts equally, but at a more coronal level—at the platform level—where the connective fibers are retained. It could be hypothesized that the rehabilitation macro-geometry dictates soft and hard tissue position, independent of the effect of the inflammatory infiltrate produced by the gap [10,11].

The gingivally convergent abutment was developed with the idea of maximizing the available space for soft tissues, which is occupied by the bulky metal shoulder in divergent abutments [12]. The sloping profile of gingival convergent abutments would allow tissue to slide coronally in the early phases of healing, creating a thick connective seal above the IA gap.

What is really bearing the brunt of preserving marginal bone levels? Is it either the relative location of the implant-abutment (IA) junction or is it the connection macro-geometry?

The specific aim of this cohort study was to investigate the clinical and radiological outcome of implants with a convergent implant-abutment connection one year after loading.

## 2. Materials and Methods

This study was a retrospective, non-interventional analysis of consecutive patients treated with dental implants with a gingivally convergent abutment connection (Shelta XA, Sweden & Martina, Via Veneto 19, 35020, Due Carrare, Padova, Italy). This study was based on patients consecutively treated on a routine basis at one specialistic center (BORG, Carrer de la Mare de Déu de Sales, 67 08840 Viladecans, Barcelona, Spain) during the period from 2016 to 2018.

### 2.1. Inclusion and Exclusion Criteria 

The medical records of patients who had at least one two-part implant rehabilitated with a convergent abutment with a one-year follow-up were reviewed. Patients were included if presenting a complete set of follow-up radiographs and intra-oral digital photographs. All implants were placed at a slightly sub-crestal level. Patient records were excluded if they did not present for bi-annual follow-up visits, if they had been rehabilitated with overdentures or full-arch prosthesis or if the implants had been placed with simultaneous guided bone regeneration.

### 2.2. Data Collection and Analysis

Data were directly entered into an Excel spreadsheet and then converted to a .csv file format in order to be read by the software for statistical analysis. The following population describing the variables were collected: Age, gender, implant characteristics (diameter, length), implant location (tooth number and anterior/posterior, maxillary/ mandibular), type of implant-supported prosthetic restoration (single crown or partial bridge), and follow-up time. 

### 2.3. Radiologic Marginal Bone Level Evaluation

**Figure 1:**
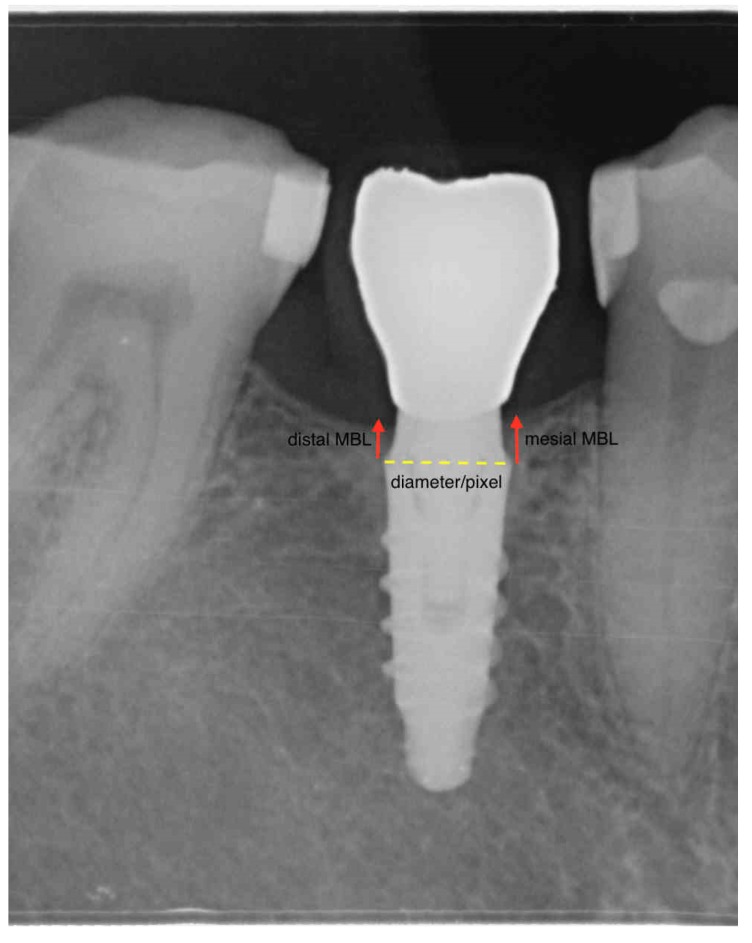
The picture is a schematic representation of the calibration performed on the software to achieve bone level linear measurements. The scale was set and calibrated by the width of the dental implant.

Routine peri-apical radiographs obtained via the long-cone paralleling technique with a loop film holder (Rinn, Dentsply Australia Pty Ltd, Pacific Hwy, St Leonards NSW 2065, Australia) were used to measure the marginal bone levels. Radiographs were standardized by means of individual resin bites. The distance between the implant–abutment connection and the first bone-to-implant contact (fBIC) on mesial and distal surfaces was recorded. The scale was calibrated by the width of the dental implant achieving a unique pixel/mm ratio (Figure 1). Radiographic bone levels were calculated at the moment of prosthetic transfer connection (impression taking), at loading, and every six months after loading. The mean marginal bone level for each implant was computed merging mesial and distal variations. The marginal bone change was defined as the difference between the last follow-up and the baseline MBL value, with negative values denoting a loss in bone height.

All measurements were performed by a single examiner (SM). The intra-examiner reproducibility was evaluated using the intraclass correlation analysis from the measurements in 10 patients, which revealed a strong correlation coefficient of 0.982 for MBL radiological measurements. Measurements were performed via the OsirisX software (Pixmeo SARL, 266 Rue de Bernex, CH-1233 Bernex, Switzerland).

### 2.4. Dichotomous Outcomes

- Implant failure was identified with eventual implant mobility or persistent infection, and whenever the implant presented signs and symptoms that led to the implant removal.
- Survival and success rates (SRs and CSRs, respectively) for implants, were calculated according to the criteria defined by Buser et al. in 1997 [13]. Successful implants were those showing a mean radiological peri-implant bone resorption within 1.5 mm during the first year of loading, and less than 0.2 mm/year during the following years.

### 2.5. Statistical Analysis

Descriptive and longitudinal statistics was performed on the R free software version 3.5.1 (02-07-2018). The longitudinal nonparametric analysis on marginal bone levels was implemented on the ld.f1 function within the package nparLD. This non-parametric method exhibits a competitive performance for small sample sizes and outliers. In the per-implant analysis, the ANOVA-type statistic (ATS) was calculated for the global alternatives with ‘time’ as the fixed su-plot factor. A *p* value < 0.05 has been used as a cut-off for significance and a robust analysis of variance and a Spearman’s correlation coefficient has been performed. A further mixed effect model (function lmer within package lme4) was used to control for crossed random effects posed by patients contributing with more than one implant. This formula expects that there is going to be multiple responses per patient, and these responses will depend on each subject’s baseline level. This effectively resolved the non-independence that stemmed from having multiple responses by the same subject.

## 3. Results

### 3.1. Study Population

In total, 20 patients received 36 implants. The mean age at the implant insertion was 56.2 ± 10.2 years (Table 1). Of the 20 patients, 65.0% were female and 35.0% were male. Of the 36 implants, 24 (66.6%) were placed in the maxilla and 12 (33.3%) were placed in the mandible. Implant diameters ranged from 3.8 mm to 5.0 mm—the mode being 4.2 mm diameter (70%)—and implant lengths ranged from 8.5 mm to 15 mm. Sixteen implants (44.4%) were splinted. Implants were more frequently placed in upper premolar positions (60%). Abutment heights ranged from 4 mm to 6 mm.

### 3.2. Survival and Adverse Events 

At the last follow up, all 36 implants were healthy, stable and there were no reported failures; thus, the implant had a cumulative survival rate of 100%. No failure, defined as signs and symptoms that led to the implant removal, could be recorded. Therefore, the cumulative success rate was 100%. The average follow-up period was 1.5 years after loading. 

### 3.3. Bone Levels

All the implants were radiographically examined by one author alien to the treatment procedure (SM) with the OsiriX DICOM viewer (Pixmeo SARL, 266 Rue de Bernex, CH-1233 Bernex, Switzerland).

**Figure 2:**
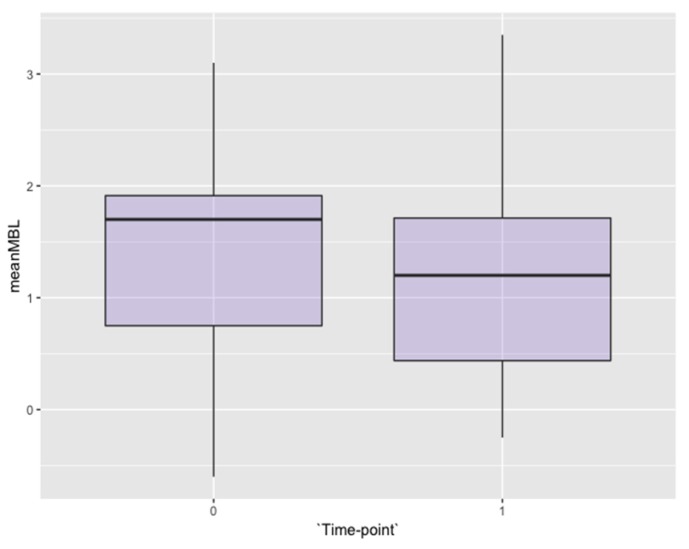
Box-plot of the mean marginal bone levels at the baseline and one year after loading.

**Figure 3:**
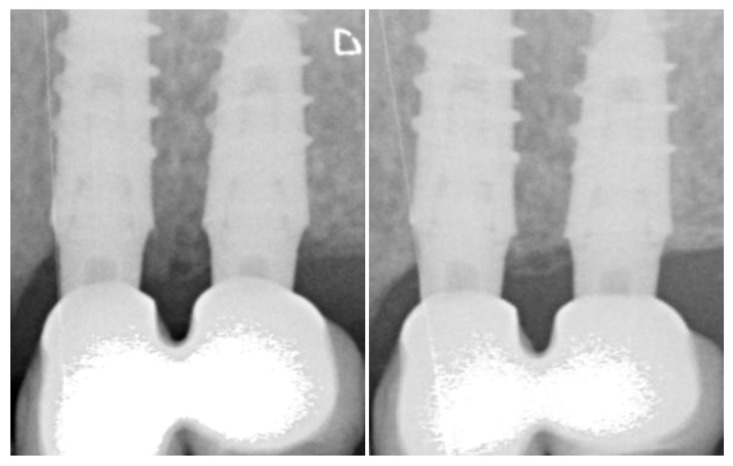
Radiographic appearance of the marginal bone levels at adjacent implants loaded with tapered abutment at loading (left) and one year after (right).

**Figure 4:**
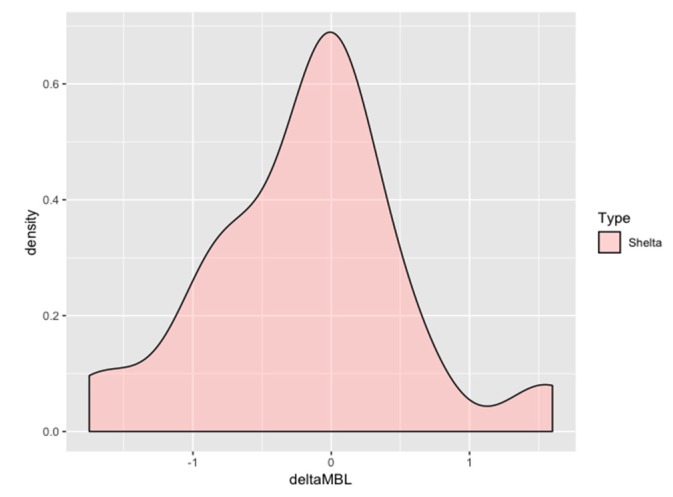
Density plot of the mean marginal bone change frequency distribution one year after loading in the entire cohort. The plot exquisitely shows a bell-shaped curve denoting a predictable amount of marginal bone resorption for the implant-abutment studied: most of the observations converged around zero.

The mean marginal bone level was +1.39 ± 0.91 mm at the moment of the prosthetic-transfer connection for definitive impression-taking (considered as the study baseline, Figure 2). One year after loading, the mean marginal bone level reached +1.16 ± 0.911 mm (Figure 2) with an average overall change of −0.18 ± 0.72 mm, occurring above the platform level at large (Figure 3). The change over time was significant (*p* value = 0.01) when the implant was modeled as the first cluster of analysis and the time was set as the only sub-plot factor (Table 2). The fitness of the model has been confirmed also with the mixed-effects model considering the random effect posed by patients contributing with more than one implant. The mean amount of bone resorption to be expected one year after loading was normally distributed (Figure 4).

The categorical data describing the implant-related factors and position (diameter, length, abutment height, jaw) were modeled on the multiway test. The implant diameter and length did not appear to affect the marginal bone, however, there was a relative significant effect given by the abutment height (*p* value < 0.05) in the mixed model: Longer abutments showed better marginal bone preservation at a one-year evaluation (Table 3). To investigate the question about which of the three abutment height categories differed, multiple comparisons with the Bonferroni adjustment were applied. The relationship held only for abutments longer than 5 mm; still, the linearity could not be confirmed. 

Implants placed in the mandible and in the maxilla did not differ in terms of 1-year marginal bone loss, however, the first bone-to-implant contact at implants placed in the mandible was significantly lower than that of the maxilla most of the times (*p* value < 0.001).

### 3.4. Secondary Outcomes: Soft Tissues

**Figure 5:**
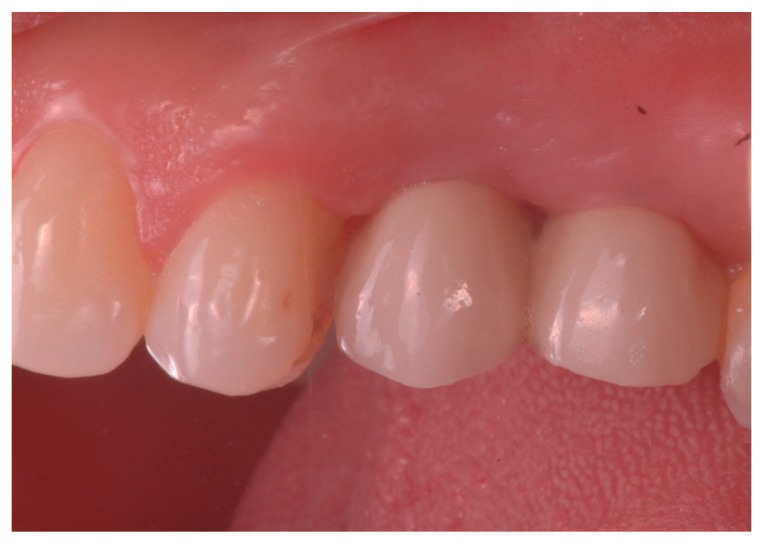
Clinical appearance of vestibular and inter-proximal soft tissues around adjacent implants loaded with tapered abutment.

Peri-implant soft tissues appeared healthy and thick at each visit after loading (Figure 5). At the provisional prosthesis loading, 96% and 95% of the 36 implants had a papilla index >2 for the mesial and distal side, respectively. At the last follow up, 97% and 94% of the implants had a papilla index >2 for the mesial and distal side, respectively. 

## 4. Discussion

In the present study, the one-year healing around two-part implants with a gingival convergent abutment profile was evaluated. In 90% of the implants analyzed, the radiological bone level extended coronal to the IA border, at the abutment level. It is suggested that bone preservation may occur coronal to the IA connection of two-part implants loaded with convergent abutment profile as a consequence of advantageous macro-geometry, independent of the effect of the inflammatory infiltrate at the gap, and of the establishment of biologic width after prosthesis connection.

Results derived from animal studies showed that marginal bone resorption of about 2 mm occurred around two-part implants [14,15]. However, Welander et al. [16] suggested that osseointegration could occur coronal to the IA junction of two-part implants when the fixture was placed 2 mm sub-crestally.

A number of factors, according to prevalent literature, might influence the first-year marginal bone loss, such as neck configuration, surgical trauma, occlusal overload, mucositis, micro-gap colonization, biologic width formation, and flapless or flapped procedures [17,18,19]. The stability of the marginal bone levels might be determined by other factors, different from those acting during the healing phase. One of these factors is represented by the apico-coronal location of the implant head in respect to the bone crest [20]. In a recent systematic review, it was assessed that the effect of the sub-crestal implant positioning compared with equi-crestal position on the bone and soft tissues around dental implants with platform switching design: The authors reported that platform switch implants placed in a sub-crestal position had shown less marginal bone resorption when compared to implants placed with their head at the crest level [21].

The radiographic observation of post-loading bone remodeling generally coincided with the level of the first thread, and some authors suggested that this would be a consequence of the soft tissue’s attempt to sit on top of the dental implant creating a mechanical protective seal [22]. 

Davarpanah [23] also observed that bone resorption around the implants placed at the supra-crestal level was less than that of the implants placed at the crestal level. However, it is true that when the thread is moved in a coronal direction, the implant platform is moved upward as well. For this reason, it would be impossible to demonstrate the relative influence of each contributing factor on bone resorption. Flores-Guillen et al. [24] compared submerged and trans mucosal platform switch implants and found that there were no differences at a five-year evaluation in terms of marginal bone loss achieving a mean value of −0.73 ± 0.81 mm. Therefore, the cumulative screening literature suggested that platform switching or, more in general, connection macro-geometry is more critical than the relative position of the platform crest module in determining early bone remodeling. 

The recent systematic review by Messias et al. suggested that reporting the marginal bone change is insufficient for the correct evaluation of the implant performance: The authors recommended to report the crestal bone levels, in particular where no data is provided relative to the healing period [25]. Furthermore, reporting at which level the crestal bone is in an intimate contact with the implant seemed reasonable and more convenient for describing the effect of the IA macro-geometry on the marginal bone. In the present study, the overall bone change was −0.18 ± 0.72 mm one year after loading, occurring above the platform level, in any case. In fact, the one-year mean bone level was +1.16 ± 0.91 mm with a significant difference between the lower and upper jaw. The mean bone gain from the baseline to the last follow-up occurred in 33.0% of the implants analyzed, which is twice the frequency observed by Flores-Guillen et al. in the platform switch implants in the same given period [24].

Few studies evaluated the tissue response around the tapered convergent abutments [26]. The use of the tapered abutments, not only could improve the peri-implant bone level, but also diminish the sulcus length. In fact, it has been suggested that the biological phenomenon of the peri-implant bone preservation would be related with the circular connective tissue fibers stabilization around the abutment and the presence of a shallow sulcus [27]. In the present study, the cumulative implant success rate was 100%, with no implant showing any sign or symptom of mucositis or prosthetic complication. Peri-implant mucosa appeared healthy-pink, thick, and firm at each visit after loading. The plausible biologic explanation should be sought in the wound healing process that starts after the abutment connection: The convergent abutment would create a housing effect that protects the surrounding biological structures maintaining tissue stability over time.

The multiway analysis conducted on this study displayed a significant relative effect of the abutment height on the marginal bone loss: Implants with longer abutments (>5 mm) appeared to have minimal bone resorption. It has been hypothesized that an abutment with a height <2 mm does not provide sufficient soft tissue for establishing the peri-implant biologic width [28]. The establishment of the peri-implant biologic width follows the implant placement and connective tissue attachment to the abutment. Long abutments might be associated with a thicker gingival biotype, which in turn would be more effective at preventing inflammatory infiltration.

The present cohort study has different limitations that should be taken into account. First, the study design was retrospective, and a single-cohort, thus reducing the meaningfulness and external validity of the results. The implant was chosen as the first cluster of analysis which does not guarantee independence between implants, however the mixed effect model applied took the random effect posed by patients into account, not revealing any significant discrepancy with the fixed effect model. It must be remarked that the radiographic artifact of a stable first bone-to-implant contact does not necessarily imply histologic osseo-integration. However, the imaging accuracy of digital radiography is high with a precision of 0.1 mm or less. Still, the clinical relevance of such small entities is questionable and difficult to repeat among different operators [29]. Furthermore, the present study is a single cohort study without an internal control group. 

## 5. Conclusions

Overall, the present study showed that implants rehabilitated with tapered abutments yielded excellent hard- and soft-tissue outcomes. In particular, after one year of loading, marginal bone levels consistently appeared above the implant platform, at the abutment level, with minimal bone change. It was suggested that the implant-connection macro-geometry might dictate peri-implant bone levels. Therefore, further prospective randomized trials are strongly recommended to support the present findings.

## Figures and Tables

**Table 1:** Demographic data and clinical characteristics.

	Male	Female	Total
Number of Patients	7	13	20
Number of Implants	9	27	36
Mean Age			56.2 ± 10.2
Age Range			39–76

**Table 2:** Mean marginal bone level (MBL) in function by year, mm (per implant analysis) and statistical significance of time-effect according to the Behrens-Fisher test and the ANOVA results for implant-related factors.

Time-point	Mesial MBL	Distal MBL	Mean MBL	Delta MBL	*p*-value
Mean MBL in function by year, mm (per implant analysis) and statistical significance of time-effect.					
**Overall**					
**Baseline**	1.47 ± 0.87	1.30 ± 1.01	1.39 ± 0.91		
**1-year**	1.28 ± 0.98	1.04 ± 0.92	1.16 ± 0.91	−0.18 ± 0.72	0.01
**Mandible**					
**Baseline**	0.90 ± 0.76	0.55 ± 0.87	0.72 ± 0.77		
**1-year**	0.76 ± 0.67	0.47 ± 0.61	0.61 ± 0.60	−0.10 ± 0.29	0.19
Sub-plot factor analysis for “*Mandible* vs. *Maxilla* relative *treatment effect”* <MBL ~ jaw *p*-value 6.58 × 10^−7^					
**Maxilla**					
**Baseline**	1.72 ± 0.81	1.63 ± 0.89	1.68 ± 0.72		
**1-year**	1.51 ± 1.02	1.29 ± 0.92	1.40 ± 0.93	−0.22 ± 0.84	0.06

**Table 3:** Mean marginal bone level (MBL) by implant abutment-height by year.

**Sub-plot factor analysis for “*Abutment Height treatment effect”* <MBL ~ abutment height *p*-value 0.05**					
**Time-point**	**mesial MBL**	**distal MBL**	**mean MBL**	**delta MBL**	***p*-value**
**4 mm**					
**Baseline**	1.31 ± 0.96	0.97 ± 0.98	1.15 ± 0.94		
**1-year**	1.08 ± 0.91	0.77 ± 1.03	0.93 ± 0.93	−0.20 ± 0.83	0.36
**5 mm**					
**Baseline**	1.54 ± 0.86	1.52 ± 1.03	1.53 ± 0.92		
**1-year**	1.34 ± 1.08	1.16 ± 0.86	1.25 ± 0.94	−0.20 ± 0.70	0.17
**6 mm**					
**Baseline**	1.80 ± 0.69	1.43 ± 0.80	1.62 ± 0.70		
**1-year**	1.87 ± 0.35	1.53 ± 0.56	1.70 ± 0.43	0.08 ± 0.34	0.05
